# Evidence-based clinical practice guidelines for the management of acute ankle injuries according to: a PRISMA systematic review and quality appraisal with AGREE II

**DOI:** 10.1186/s12891-024-07655-z

**Published:** 2024-07-08

**Authors:** Najeeb Bsoul, Liang Ning, Leyi Cai, Davit Mazmanyan, Daniel Porter

**Affiliations:** 1 Tsinghua University, Beijing, China; 2 First Affiliated Hospital of Tsinghua University, Beijing, China; 3 Second Affiliated Hospital of Wenzhou Medical University, Wenzhou, China

**Keywords:** Acute, Ankle, Injury, Evidence-based, Clinical practice, Guideline, AGREE II

## Abstract

**Background:**

Acute ankle injuries are commonly seen in emergency rooms, with significant social impact and potentially devastating consequences. While several clinical practice guidelines (CPGs) related to ankle injuries have been developed by various organizations, there is a lack of critical appraisal of them. The purpose of this systematic review is to identify and critically appraise evidence-based clinical practice guidelines (EB-CPGs) related to acute ankle injuries in adults.

**Method:**

We conducted searches in the Cochrane Library, MEDLINE, EMBASE databases, WHO, and reviewed 98 worldwide orthopedic association websites up until early 2023. Two authors independently applied the inclusion and exclusion criteria, and each evidence-based clinical practice guideline (EB-CPG) underwent independent critical appraisal of its content by all four authors using the Appraisal of Guidelines for REsearch and Evaluation (AGREE II) instrument. AGREE II scores for each domain were then calculated.

**Results:**

This review included five evidence-based clinical practice guidelines. The mean scores for all six domains were as follows: Scope and Purpose (87.8%), Stakeholder Involvement (69.2%), Rigour of Development (72.5%), Clarity of Presentation (86.9%), Applicability (45.6%), and Editorial Independence (53.3%).

**Conclusion:**

The number of EB-CPGs related to ankle injuries are limited and the overall quality of the existing evidence-based clinical practice guidelines (EB-CPGs) for ankle injuries is not strong, with three of them being outdated. However, valuable guidance related to Ottawa rules, manual therapy, cryotherapy, functional supports, early ambulation, and rehabilitation has been highlighted. Challenges remain in areas such as monitoring and/or auditing criteria, consideration of the target population’s views and preferences, and ensuring editorial independence. Future guidelines should prioritize improvements in these domains to enhance the quality and relevance of ankle injury management.

**Systematic review:**

Systematic review.

**Supplementary Information:**

The online version contains supplementary material available at 10.1186/s12891-024-07655-z.

## Background

Acute ankle injury ranks among the most frequently encountered musculoskeletal [1, 2] and intra-articular injuries [3, 4]. Specifically, it stands out as the predominant lower limb injury in individuals engaged in physical activities [5]. In Western countries, nearly one ankle sprain occurs each day per 10,000 individuals [6, 7]. The incidence rate is 4.22 ankle fractures per 10,000 person-years within the U.S. population [8]. Consequently, emergency departments in the United States and the United Kingdom collectively treat over two million cases each year [1, 9]. Ankle injuries can manifest due to a diverse range of mechanisms [10, 11], and reports have documented associations between falls, jumps, sports, and various forms of trauma as contributors to such injuries [1, 11–15]. Ankle injuries exhibit higher prevalence rates among specific demographic groups, including females, children, and athletes engaged in indoor and court sports [2, 7]. In the context of sports, the incidence of ankle injuries constitutes a substantial portion, accounting for 16–40% of all sport-related trauma [7, 16]. Approximately 40% of all traumatic ankle injuries occur during athletic activities [17]. Notably, among these pursuits, basketball (41.1%), American football (9.3%), and soccer (7.9%) stand out as having the highest incidence rates [5, 7, 17]. In a study encompassing a population of 8,092,281 soldiers from 15 different countries, a total of 788,469 foot and ankle injuries were documented, constituting approximately 9.74% of all injuries [7, 18]. The acute phase refers to the period within two weeks following the injury [19, 20]. Pain, swelling and stiffness are the most common signs of acute ankle injuries. Typically, these symptoms subside within six weeks, yet they may persist for an extended duration [21]. Complications arising from acute ankle injuries can lead to significant costs and encompass conditions such as chronic ankle instability (CAI) [22, 23], post-traumatic ankle osteoarthritis (PTOA) [24], and an elevated risk of falls among older populations [25].

The management of acute ankle injuries can involve a range of healthcare professionals, including orthopedic surgeons, sports physicians, physical therapists, athletic trainers, nurse practitioners, school nurses, general practice physicians, emergency room staff (doctors, nurses, and physical therapists), community pharmacists (pharmacists, pharmacy assistants, and shop assistants), as well as first aid officers [26–28]. The effectiveness of their involvement in the management process is contingent on their educational background, training, ongoing professional development, and adherence to evidence-based clinical practice guidelines (EB-CPGs) [29]. Evidence-based guidelines, often referred to as trustworthy guidelines, possess five key characteristics: they are based on systematic reviews of the literature, include ratings of the quality of evidence, provide the strength of recommendations, consider patient values, and are developed by a multidisciplinary panel of experts [30]. Well-crafted evidence-based clinical practice guidelines (EB-CPGs), grounded in high-quality evidence, serve as a conduit to align policy, best practices, local contexts, and patient preferences [31]. The evolution of clinical practice guideline development necessitates continuous adaptation [32, 33]. Useful insights for their creation can be gleaned from tools documentation supporting evaluation tools such as the Appraisal of Guidelines for Research & Evaluation II (AGREE II) [34].

Data related to the appraisal of EB-CPGs at a nationwide level for ankle injuries is currently limited. The availability of such information is vital for more accurately assessing the morbidity rates linked to these injuries, evaluating their financial implications, and facilitating the adoption of preventive measures. A guideline appraisal regarding ankle sprain, a subset of ankle injury, was published in 2019 [29]. The review encompassed both EB-CPGs and less rigorously designed guidance such as ‘expert consensus [29].

### Objectives

By identification and compilation of an up-to-date list of English language evidence-based clinical practice guidelines (EB-CPGs) relevant to acute ankle injuries in adults, and subsequent critical assessment of these CPGs using the Appraisal of Guidelines for Research and Evaluation (AGREE) II instrument, we aimed to identify strengths and weaknesses to assist current users and future guideline developers.

## Methods

### Protocol and registration

The search strategy, as well as the criteria for inclusion and exclusion, were pre-defined and formally documented as below:

### Database search strategy

In January 2023, we conducted an electronic search across key medical literature databases, including the Cochrane Library, MEDLINE, EMBASE, WHO, and 98 worldwide orthopedic association websites. This comprehensive approach aimed to identify all English language clinical practice guidelines (CPGs) related to acute ankle injuries. The inclusion of additional searches on professional organization websites is crucial, as not all EB-CPGs may be published as referenced articles in scientific databases.

The search strategy was developed and refined through an international collaboration within a clinical team of guideline review experts (DP, LN, NB, LC, and DM), representing both developing and developed countries in Europe and Asia. Two researchers independently conducted keyword searches related to ankle injuries (NB & LN), using a combination of medical subject headings and title search terms. The strategy underwent further refinement through team discussion.

In addition to medical databases, other sources such as Google Scholar, hand searching, and personal communication were utilized to gather relevant guidelines. Only evidence-based clinical practice guidelines (EB-CPGs) published in English were considered for retrieval. The retrieval process adhered to the PRISMA flow algorithm. All data supporting the findings of this study are available within the paper and its Supplementary Information.

### Study selection

The records retrieved from the search were imported into Endnote 20 (Thomson Reuters, New York, USA) for management [35]. Subsequently, the software was used to identify and eliminate any duplicate records from the dataset.

### Inclusion criteria

This review included evidence-based clinical practice guidelines (CPGs) designed for adults aged 18 years and older who experienced acute ankle injuries and were published in English.

### Exclusion criteria

This review excluded evidence-based clinical practice guidelines (EB-CPGs) intended for patients younger than 18 years old, as well as those addressing pathological ankle injuries, open fractures, distal tibial metaphyseal fractures (pilon fractures alone), and chronic injuries. Additionally, CPGs published in languages other than English, those not developed by a group of experts, or those solely based on a single series study or developed without evidence-based methodology were not considered in the review.

### Data collection

The inclusion of studies was determined by four researchers (DP, LN, NB, LC, and DM), who independently assessed the title, abstract, and full text of the identified sources. Any disagreements were resolved through discussion involving the senior author (DP).

The search for guidelines was independently conducted by two researchers (NB and LN) using the database websites up to January 1, 2023. The guideline search utilized the keywords obtained through the aforementioned process. Both keyword-based and manual searches were carried out. The process of retrieving guidelines followed the Preferred Reporting Items for Systematic Reviews and Meta-Analyses (PRISMA) flow algorithm recommendations and was consistent with the methodology of a systematic review of acute ankle sprain guidelines [29, 36].

### AGREE II data collection process

The Appraisal of Guidelines for Research and Evaluation II (AGREE II) [34] is a standardized and globally recognized tool used for critically appraising clinical practice guidelines (CPGs). Developed specifically to address variability in CPG quality, AGREE II provides a structured and systematic process for evaluating methodological rigor, transparency in CPG development, and quality of reporting. AGREE II comprises 23 items grouped into six domains: scope and purpose (3 items), stakeholder involvement (3 items), rigour of development (8 items), clarity of presentation (3 items), applicability (4 items), and editorial independence (2 items). Each item is assessed on a seven-point scale, ranging from 1 (strongly disagree) to 7 (strongly agree).

Appraisers also have the opportunity to provide an overall evaluation of the guideline’s quality on the same seven-point scale, where 1 represents the lowest quality and 7 indicates the highest. Additionally, appraisers can indicate whether they would recommend the guideline for use, with response choices including “Yes,” “Yes with modifications,” or “No.”

The AGREE II methodology involves calculating domain scores by summing up the scores of individual items within each domain and scaling the total as a percentage of the maximum possible score for that domain. This structured approach enables the assessment and comparison of clinical practice guidelines across different domains.

AGREE II has undergone rigorous testing for both validity [37] and reliability [33], with the results published in peer-reviewed journals. These studies consistently confirm AGREE II’s validity and reliability, demonstrating sufficient inter-rater reliability.

Endorsed by two systematic reviews [38, 39], AGREE II is recognized as the only validated tool for evaluating evidence-based clinical practice guidelines (EB-CPGs). It allows for the calculation of a numerical score, providing a semi-quantitative evaluation of EB-CPGs, their domains, and items.

To enhance inter-rater reliability, AGREE II recommends the involvement of at least two, ideally four, appraisers in assessing a single practice guideline. In this study, four researchers (DP, LN, NB, DM) conducted the appraisal. All authors were familiar with the AGREE II user manual and had received training through online tutorials available on the AGREE II website (http://www.agreetrust.org/agree-ii/).

### Statistical analysis

The scoring process involved calculating a score for each domain by summing the scores of all the individual items within that specific domain. The score was then standardized using the following formula: (obtained score - minimum possible score) / (maximum possible score - minimum possible score).

For individual items within the domains, the same scoring method was applied as for the domain scores. The primary purpose of the AGREE scale is to emphasize and promote best practices. Therefore, our approach was to focus detailed critique efforts on domains that met an ‘acceptable’ quality threshold.

Following convention in existing research reports, the domain score criteria we used were as follows: <40% very low quality, 40%~59% low quality, 60%~79% acceptable quality, and ≥ 80% good quality [40, 41].

For the overall assessment of guidelines, scores provided by appraisers for item 1 in the ‘overall assessment’ section were used to compute a score for each guideline. The same standardization method applied to domain scores was used for this calculation [42].

## Results

### Study selection

**Fig. 1:**
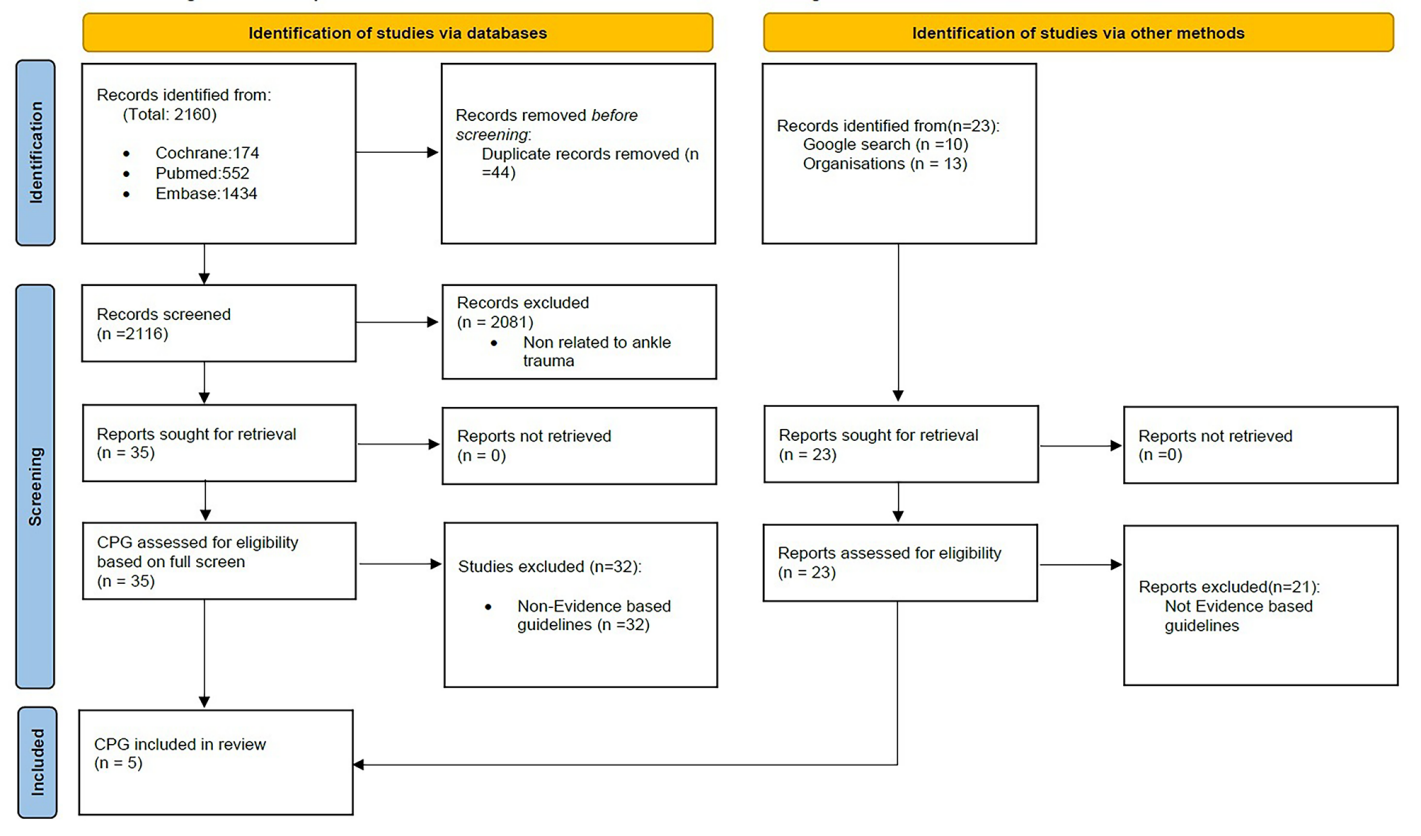
PRISMA flow diagram. PRISMA = preferred reporting items for systematic reviews and meta-analyses

From the database search, a total of 2160 articles were exported to Endnote 20 [35], with distribution as follows: Cochrane database: 174 articles; Medline database: 552 articles; Embase database: 1434 articles. After removing 44 duplicates, 2081 articles were excluded following screening of the title and abstract content. The full text of the remaining 35 articles was obtained and screened according to the inclusion and exclusion criteria of this study, resulting in the exclusion of 32 articles due to being non-evidence-based guidelines. Consequently, a total of 3 guidelines for ankle trauma were identified from the database searches (Fig. 1). These comprised two EB-CPGs from the American College of Radiology [43] and the American Physical Therapy Association (updated) [22], along with one from the Royal Dutch Society for Physical Therapy Dutch (updated) [44].

A total of 23 guidelines were retrieved by searching the websites of reputable international professional organizations/associations with interests in lower-limb trauma and ankle disorders, WHO, and a manual search on Google. Following full-text screening, an additional total of 2 clinical guidelines for ankle trauma were obtained through Orthopedic associations based in the United Kingdom [45] and the Netherlands [46] (Fig. 1). Table 1 lists the year of publication, title, authorship, and target user group for these five EB-CPGs. Excluded guidelines with reasons highlighted are provided in the supplementary material.

**Table 1:** Description of the acute ankle injuries clinical practice guidelines

Number	Publication date	Name	Author	Target Health Professional
1	2021	Ankle Stability and Movement Coordination Impairments: Lateral Ankle Ligament Sprains Revision 2021	Martin, R. L., et al. [42]	Physical therapists who are members of the American Physical Therapy Association
2	2020	ACR Appropriateness Criteria Acute Trauma to the Ankle.	Expert Panel on Musculoskeletal Imaging [43]	Orthopedic surgeons, emergency doctors, and radiologists
3	2018	Diagnosis, treatment, and prevention of ankle sprains: update of an evidence-based clinical guideline	Kerkhoffs, G. M., et al. [40]	Physical therapists, orthopaedic and trauma surgeons, family, rehabilitation, occupational, and sports physicians, radiologists, and professionals involved in sport massage
4	2015	Suture fixation of acute disruption of the distal tibiofibular syndesmosis	NICE [45]	Orthopedic surgeons in UK
5	2006	KNGF Guideline for Physical Therapy in patients with acute ankle sprain - Practice Guidelines	Wees, P., et al. [37]	Physical therapists who are members of the Royal Dutch Society of Physical Therapy

### AGREE II analysis

**Fig. 2:**
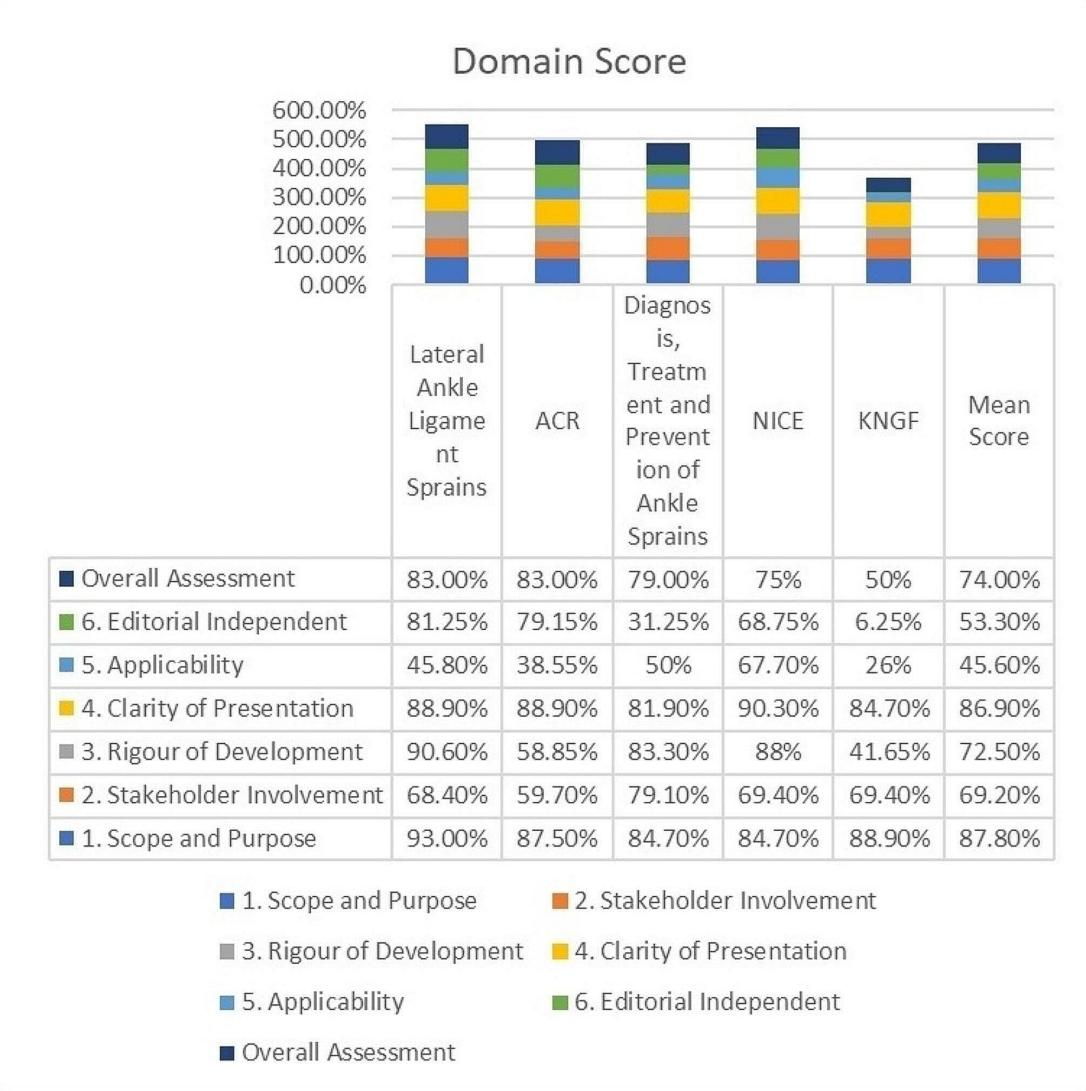
Summated AGREE II domain scores from each clinical practice guideline related to acute ankle injury

The AGREE II scores, calculated as a percentage of the maximum possible score and obtained from the assessments of the four independent reviewers, are depicted in Fig. 2. Mean domain scores are presented in the same figure. Item scores can be found in Table 2.

**Table 2:** Categories of clinical practice relating to acute ankle injuries that are or are not covered by these five evidence-based clinical practice guidelines

Area/Type of Injury	Sprain	Dislocation	Fracture
A) Diagnosis			
- Physical Examination	Yes	Yes	-
- Imaging	Yes	Yes	yes
B) Treatment			LAF related
- Conservative	Yes	-	-
- Surgical	DFTS related	-	-
- Drugs	Yes, but not well-covered	-	-
C) Follow up: Physio/Rehab	Yes	-	-
D) Prevention	Yes	-	-
E) First Aid	-	-	-

Legend: LAF = Lateral Ankle Fracture; DTFS = Distal Tibiofibular Syndesmosis; Physio = Physiotherapy; Rehab = Rehabilitation

### Domains with mean scores exceeding 80% (good quality)

the ‘Scope and Purpose’ domain achieved a mean score of 87.8%, consistently exceeding 80% across all guidelines. Each of the three items contributing to this domain received mean scores surpassing 80%. Similarly, the ‘Clarity of Presentation’ domain attained a mean score of 86.9%, with all guidelines scoring above 80%. All three items within this domain also received mean scores above 80%.

### Domains with mean scores ranging from 60 to 79% (acceptable quality)

The ‘Stakeholder Involvement’ domain achieved a mean score of 69.2%, with scores ranging from 59.7–79.1% across the guidelines. However, item five scored below 60% in each of the five guidelines evaluated. Conversely, the mean score for the ‘Rigour of Development’ domain reached an acceptable level of 72.5%. Notably, guidelines for Lateral Ankle Ligament Sprains, Diagnosis, treatment, and prevention of ankle sprains, and NICE scored above 80% in this domain, while the KNGF and ACR guidelines scored below 60%. In the KNGF guideline, items 7, 13, and 14, and in the ACR guideline, item 9, scored below 40% [43, 46].

### Domains with mean scores ranging from 40 − 59% (low quality)

In the ‘Applicability’ domain, the mean score was 45.6%. The ACR and KNGF guidelines scored below 40% in this domain, while the NICE guideline scored above 59%. Items 18 to 20 had a mean score ranging from 40 to 60%. Conversely, item 21 scored below 40%. Finally, regarding ‘Editorial Independence,’ it attained a mean score of 53.3%, with a wide variation in the quality of this domain between the guidelines, ranging from 6.3 to 81.25%. KNGF and Diagnosis, treatment, and prevention of ankle sprains guidelines scored below 40% in items 22 and 23. [37, 40].

## Discussion

The primary aim of this systematic review is to identify and critically evaluate all English-language evidence-based clinical practice guidelines (EB-CPGs) regarding the management of acute ankle injuries in adults using the AGREE II instrument. We identified five EB-CPGs that met our inclusion criteria, covering important areas of clinical practice. However, large areas of practice have yet to be addressed by CPG committees (Table 3). Furthermore, even among the published guidelines, significant deficiencies exist.

**Table 3:** AGREE II items’ scores and the mean score for each guideline

Item	Lateral Ankle Ligament Sprains	ACR	Diagnosis, Treatment and prevention of Ankle Sprains	NICE	KNGF	Mean
Score & Purpose						
1. The overall objective(s) of the guideline is (are) specifically described.	100%	87.5%	91.7%	87.5%	87.5%	90.8%
2. The health question(s) covered by the guideline is (are) specifically described.	87.5%	87.5%	83.3%	83.3%	87.5%	85.8%
3. The population (patients, public, etc.) to whom the guideline is meant to apply is specifically described.	91.7%	87.5%	79.2%	83.3%	91.7%	86.7%
Stakeholder involvement						
4. The guideline development group includes individuals from all the relevant professional groups.	79.2%	79.2%	83.3%	79.2%	83.3%	80.8%
5. The views and preferences of the target population (patients, public, etc.) have been sought.	50%	33.3%	58.3%	54.2%	29.2%	45%
6. The target users of the guideline are clearly defined.	75%	66.7%	95.8%	75%	95.8%	81.7%
Rigour of development						
7. Systematic methods were used to search for evidence.	100%	70.8%	95.8%	95.8%	8.3%	74.1%
8. The criteria for selecting the evidence are clearly described.	100%	79.2%	95.8%	95.8%	45.8%	83.3%
9. The strengths and limitations of the body of evidence are clearly described.	91.7%	37.5%	75%	83.3%	58.3%	69.2%
10. The methods for formulating the recommendations are clearly described.	79.2%	75%	91.7%	75%	50%	74.2%
11. The health benefits, side effects, and risks have been considered in formulating the recommendations.	70.8%	62.5%	66.7%	100%	70.8%	74.2%
12. There is an explicit link between the recommendations and the supporting evidence.	87.5%	50%	83.3%	100%	87.5%	81.7%
13. The guideline has been externally reviewed by experts prior to its publication.	95.8%	50%	66.7%	83.3%	4.2%	60%
14. A procedure for updating the guideline is provided.	100%	45.8%	91.7%	70.8%	8.3%	63.3%
Clarity of presentation						
15. The recommendations are specific and unambiguous.	83.3%	87.5%	83.3%	91.7%	87.5%	86.7%
16. The different options for management of the condition or health issue are clearly presented.	87.5%	87.5%	87.5%	87.5%	87.5%	87.5%
17. Key recommendations are easily identifiable.	95.8%	91.7%	75%	91.7%	79.2%	86.7
Applicability						
18. The guideline describes facilitators and barriers to its application.	33.3%	29.2%	62.5%	54.2%	20.8%	40%
19. The guideline provides advice and/or tools on how the recommendations can be put into practice.	75%	45.8%	58.3%	91.7%	29.2%	60%
20. The potential resource implications of applying the recommendations have been considered.	45.8%	54.2%	45.8%	66.7%	16.7%	45.8%
21. The guideline presents monitoring and/or auditing criteria.	29.2%	25%	33.3%	58.3%	37.5%	36.7%
Editorial independence						
22. The view of the funding body have not influenced the content of the guideline.	83.3%	75%	25%	66.7%	4.2%	50.8
23. Competing interests of guideline development group members have been recorded and addressed.	79.2%	83.3%	37.5%	70.8%	8.3%	55.82%

All five EB-CPGs evaluated in our study are relevant to soft tissue injuries of the ankle [22, 43–46]. However, despite the significant global burden of acute ankle injuries, we were unable to identify any EB-CPGs specifically focusing on the management of this injury in the emergency setting. Additionally, while first aid officers play a crucial role in providing essential assistance and early care to individuals with acute ankle injuries during work, sports, and public events [47], there are no EB-CPGs designed for use by pre-hospital practitioners. In countries like Australia and New Zealand, pharmacists follow the RICE (rest, ice, compression, elevation) protocol, recommend NSAIDs, and utilize handbooks to inform their decision-making for acute lateral ankle ligament sprain (LALS) [48–50]. However, EB-CPGs for this specific context are lacking. Furthermore, in the realm of orthopedic fracture management, EB-CPGs were deficient in important acute diagnostic and management categories, such as disruptions to the distal tibiofibular syndesmosis, injuries to the medial (deltoid) ligament, or management of ankle fractures in emergency room settings.

Among the existing EB-CPGs we reviewed, none achieved consistently high scores across all domains. “Ankle Stability and Movement Coordination Impairments: Ankle Ligament Sprains,” associated with the International Classification of Functioning, Disability, and Health from the orthopedic section of the American Physical Therapy Association [22], received the highest overall score across all domains, closely followed by the NICE guideline [45]. Conversely, the KNGF guideline for physical therapy in patients with acute ankle sprain [46] received the lowest overall score, which is consistent with its early development date (published in 2006). These findings highlight variations in the quality and comprehensiveness of the EB-CPGs, with certain domains demonstrating higher overall quality while others reveal potential deficiencies (Fig. 2), suggesting areas for improvement in guideline development and presentation.

### Domains with mean scores exceeding 80% (good quality)

Domains “Scope and Purpose” and “Clarity of Presentation” and their items were generally strong in all EB-CPGs assessed (ranging from 84.7 to 93.0% and 81.9–90.3%, respectively; see Fig. 2), indicating that these CPGs generally had clear objectives and were presented in a comprehensible manner.

### Domains with mean scores ranging from 60 to 79% (acceptable quality)

Overall, four domains exceeded the threshold for ‘acceptable’ quality (mean score of 60% or above): ‘Scope and Purpose’, ‘Stakeholder involvement’, ‘Rigour of development’, and ‘Clarity of presentation’.

While the overall score of guidelines in the “Rigour of Development” domain is acceptable, the KNGF and ACR guidelines score below 60% in specific items. For example, in the KNGF guideline, items 7, 8, 9, 10, 13, and 14, and in the ACR guideline, items 9, 12, 13, and 14, had scores below 60%. We emphasise that external review by experts is a fundamental step in ensuring the quality and credibility of evidence-based clinical practice guidelines (EB-CPGs) prior to their publication; this step adds support where important recommendations are made despite deficiencies in the evidence-base [51, 52].

Item 5 in domain 2 (stakeholder involvement) evaluates the degree to which relevant experts, stakeholders, patients, and the public have contributed to diverse perspectives, experiences, and expertise, leading to the creation of more comprehensive, relevant, and effective CPGs. Item 5, ‘The views and preferences of the target population (patients, public, etc.) have been sought,’ had the lowest score among the three items in this domain, and all guidelines scored < 60% on this important item. The low scores reflect the absence of multidisciplinary teams and the failure to consider patients’ views and experiences during guideline development. For example, the KNGF [46] and ACR [43] guidelines did not show clear information obtained from interviews of these stakeholders or from literature reviews of patient/public values, preferences, or experiences. There were no formal consultations with patients/public to determine priority topics, participation of these stakeholders on the guideline development group, or rigorous external review by these stakeholders on draft documents. Within specific guideline projects, patients’ opinions may be incorporated when framing the question so patients should again be engaged at the developer level by helping determine when guidelines need updating and evaluating the developer’s approach to patient engagement [53].

Guideline developers need to thoughtfully consider patient engagement at each step; specific goals may dictate the types of engagement utilized [54]. Engaging patients through multiple mechanisms—such as guideline development group (GDG) participation and public comment—has advantages and may be a particularly important strategy for addressing barriers such as GDG members representing only select views [53, 55, 56].

Including professionals from various related disciplines may be perceived as complicating the process [57]. However, to improve patient care related to the guideline’s topic, all relevant disciplines should be involved in the guideline development group [58]. Guideline developers should actively reach out to relevant patient organizations early in the process to define the guideline’s scope and include patient representatives in their panels [53, 54]. For instance, NICE actively reaches out, but the organizations often don’t respond. Additionally, they should consider searching for qualitative and other types of research on patients’ values and preferences. Ensuring such engagement is crucial for enhancing the quality and acceptance of CPGs within the medical community and promoting their effective implementation in clinical practice [51].

### Domains with mean scores ranging from 40 − 59% (low quality)

‘Applicability’ and ‘Editorial Independence’ domains had mean scores falling below 60% (range 26-67.7% and 6.25 − 81.25% respectively; Fig. 2).

During the appraisal process, we found that strategies to improve guideline implementation were seldom considered. In the five evaluated EB-CPGs, applicability scores were low because developers discussed few details relating to costs, implementation barriers, or criteria for monitoring adherence. For an extended period, the prevailing assumption among guideline developers was that presenting practitioners with well-supported evidence in a structured format would automatically lead to improved performance [59, 60]. However, findings from numerous controlled trials and systematic reviews indicate that efforts to implement guidelines often fall short of significant success [61]. At best, small to moderate improvements in the care process have been found, usually not exceeding 5–10%, depending on the implementation methods used [61]. Notably, guideline impact on patient outcomes has frequently not been studied or has been difficult to obtain [32]. Applicability was also found to be the lowest-scoring domain in a recent evaluation of English language CPGs related to spine disorders [33]. Previous work by Green et al. on CPGs and expert consensus documents related to ankle sprain also classified this domain as extremely low quality [16]. Application tools should be developed, such as indicators for performance assessment, teaching materials, patient information pamphlets, or computer decision aids [34]. It is important to involve the end users in the development process to ensure local acceptance and relevance to local practice [34, 61].

**Fig. 3:**
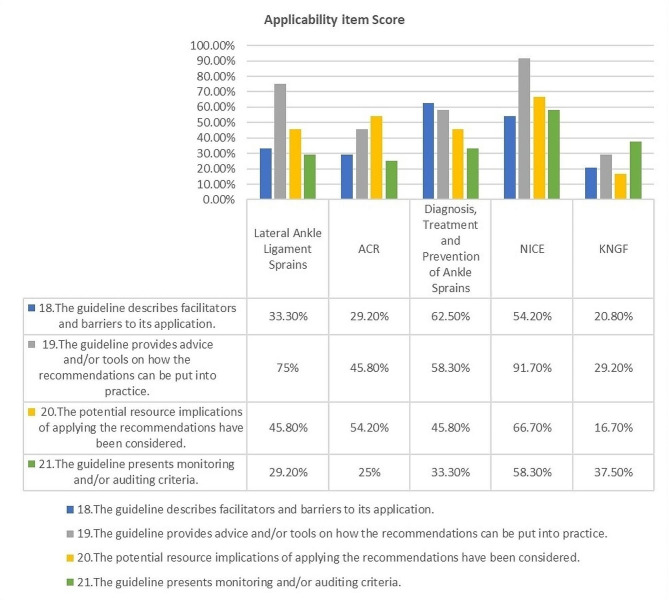
AGREE II results for domain five (Applicability)

Domain 5 (items 18–21) reviews the way in which the guideline addresses the applicability of the recommendations to end-users, including the identification of barriers and facilitators to implementation (item 18), advice and/or tools on how the recommendations can be put into practice (item 19), the potential resource implications of applying the recommendations (item 20), and the inclusion of monitoring or auditing criteria (item 21). Items 18 and 21 had the lowest overall scores of any item (see Fig. 3; Table 2).

Criteria for monitoring and/or audit purposes are frequently formulated after the publication of guidelines, often experiencing a considerable delay. This requires specialized expertise that may not be readily available during the guideline development process. Institutions involved in guideline development should consider gradually integrating these monitoring and audit considerations into the early phases of the development process [62].

Editorial Independence scored below the acceptable level in two guidelines, possibly due to insufficient information about funding sources and conflicts of interest. Improving scores in this domain could be achieved by providing more comprehensive details on these aspects. New strategies for managing financial and intellectual conflicts of interest are being introduced [63–65]. It is recommended for CPGs to include information under the following headings: Contributors, Funding, Competing Interests, Provenance and Peer Review, and Author Affiliations [64]. In the domain of editorial independence, CPGs are evaluated based on the following statements: ‘The views of the funding body have not influenced the content of the CPG’ and ‘Competing interests of members of the CPG development group have been recorded and addressed’ [34, 66]. The decision pathway itself should be more transparent and require a declaration of interest before participation [67]. The extent of conflict of interest should be linked to its scope and be sensitive to time limitations. CPG designers should have a greater focus on the wider decision-making process, rather than on expert activities. Conflicts of interest can be overcome by flexibility, enhanced status of expert witnesses, and by greater non-industry sponsorship of professional educational and research activities [68].

## Conclusions

This analysis exclusively evaluated evidence-based clinical practice guidelines (EB-CPGs), highlighting both their strengths and areas needing improvement in the development of future EB-CPGs related to ankle injuries. Notably, the study identified weaknesses in the areas of applicability, editorial independence, and certain aspects of stakeholder involvement within the assessed EB-CPGs. Furthermore, we confirmed an association between the overall evaluation scores of the guidelines and their year of publication, reinforcing the concept of dynamic, evolving processes of continuous improvement in the quality of CPGs over time. This reflects the ongoing efforts within the medical community to enhance the quality, relevance, and applicability of guidelines, ultimately benefiting patient care and clinical practice. Finally, there is a demand for additional guidelines in specific areas of ankle injuries.

### Limitations

The scope of this evaluation was limited to English language EB-CPGs, with non-English EB-CPGs excluded through the application of a language filter (refer to Additional file 1 for the Search Strategy and List of Articles). The number of retrieved guidelines was small, however this appraisal identified areas for improvement in existing guidelines. We believe that these insights will be valuable for developers in future updates and newly designed EB-CPGs to enhance their quality and effectiveness.

AGREE II remains a subjective evaluation tool. However, reliability is maximized by having four evaluators, and this remained an important principle in our methodology. Other published work in this area has often limited the number of evaluators to two.

There is no established method for deriving a consensus-based overall guideline score using AGREE II, neither through developer instructions nor prior literature. The process of performing quantitative scoring for the overall evaluation of evidence-based clinical practice guidelines (EB-CPGs) using the AGREE II tool has some variations and lacks detailed instructions in the AGREE II manual [69]. Different studies have adopted various methods to calculate overall scores, often without specific reference to the item “recommendations of the guideline for use” [70, 71]. In some studies, mean scores for the item “rate the overall quality of this guideline” have been calculated without clear guidance on how to consider the item “recommendations of the guideline for use” [70, 71]. In other cases, assessors have determined overall scores based on the average rating given to the six domains assessed by AGREE II [71, 72]. In our study, reviewers were not provided with specific instructions regarding overall recommendations, indicating that the approach to overall evaluation may have varied among assessors. This variation in scoring methods underscores the need for greater clarity and standardization in the process of evaluating EB-CPGs using the AGREE II tool, particularly in terms of assessing their overall quality and suitability for use.

### Electronic supplementary material

Below is the link to the electronic supplementary material.

Supplementary Material 1

Supplementary Material 2

## Data Availability

All data supporting the findings of this study are available within the paper and its Supplementary Information.

## Abbreviations

AGREE: Appraisal of Guidelines for REsearch and Evaluation
CPGs: evidence based Clinical practice guidelines
EB: Evidence based
CAI: Chronic ankle instability
PTOA: Post-traumatic ankle osteoarthritis
PRISMA: Preferred Reporting Items for Systematic Reviews and Meta-Analyses
Legend: LAF = Lateral Ankle Fracture; DTFS = Distal Tibiofibular Syndesmosis; Physio = Physiotherapy; Rehab = Rehabilitation
LALS: Lateral ankle ligament sprains
NSAIDs: Non-steroidal anti-inflammatory drugs
RICE: Rest, Ice, Compression, Elevation
GDG: Guideline development group
NICE: National Institute for Health and Care Excellence
KNGF: Koninklijk Nederlands Genootschap voor Fysiotherapie/ Royal Dutch Society for Physiotherapy
ACR: The American College of Radiology
