# Supplementary material for: Evidence-based clinical practice guidelines for the management of acute ankle injuries according to: a PRISMA systematic review and quality appraisal with AGREE II
Source: BMC Musculoskelet Disord. 2024 Jul 8;25:523. doi: 10.1186/s12891-024-07655-z (PMC11229291; doi:10.1186/s12891-024-07655-z)
Supplement: Supplementary file 2 — Supplementary Material 2 [file 12891_2024_7655_MOESM2_ESM.docx]

**Excluded guidelines**

| **Number** | **Guideline title** | **Reason for exclusion** | **Guideline source** |
| --- | --- | --- | --- |
| 1 | Ankle Injury – X-Ray for Acute Injury of the Ankle or Mid-Foot. | No clear information regarding methodology | <https://www2.gov.bc.ca/assets/gov/health/practitioner-pro/bc-guidelines/anklex.pdf> |
| 2 | Modified Brostrӧm-Gould Repair for Chronic Lateral Ankle Instability.  Department of Rehabilitation Services  Physical Therapy.  The Brigham and Women's Hospital, Inc., Department of Rehabilitation Services. | Not developed for acute injuries | <https://www.brighamandwomens.org/assets/BWH/patients-and-families/rehabilitation-services/pdfs/ankle-brostrom-gould-repair-for-lateral-ankle-instablility-bwh.pdf> |
| 3 | REHABILITATION AFTER ANKLE SPRAIN  Dr. Abigail R. Hamilton, MD. Twin cities orthopedics | No clear information regarding methodology | <https://www.tcomn.com/wp-content/uploads/2017/08/Ankle-Sprain-Rehab-1.pdf> |
| 4 | ANKLE OCD SURGERY REHABILITATION GUIDELINES, Laith M. Jazrawi, MD. NYU  Langone medical center. | No clear information regarding methodology | <https://www.newyorkortho.com/pdf/ankle-ocd-rehab-protocol.pdf> |
| 5 | BROSTROM PROCEDURE  CLINICAL PRACTICE GUIDELINE | Not developed for acute injuries | <https://medicine.osu.edu/-/media/files/wexnermedical/patient-care/healthcare-services/sports-medicine/education/medical-professionals/knee-ankle-and-foot/brostrom-cpg2020.pdf?la=en&hash=26BA5DF208EC168E471DA8D84218B56CD4717B85> |
| 6 | Ankle Sprains - Emergency Department. The Royal Children’s Hospital (RCH). | No clear information regarding methodology | <https://www.rch.org.au/clinicalguide/guideline_index/fractures/Ankle_Sprains_-_Emergency_Department/> |
| 7 | Clinical practice guidelines for acute lateral ankle sprain and ankle instability. | No clear methodology | <https://wrightpt.com/ankle-sprain-and-ankle-instability/> |
| 8 | Clinical Guidelines: Foot / Ankle. Orthopedic Institute of Pennsylvania. | No clear methodology | <https://www.oip.com/content/uploads/2017/03/Clinical-Guidelines-Foot-Ankle-9-26-2014.pdf> |
| 9 | Evidence Based Physiotherapy Guideline for Conservative Management of Ankle Sprain.  K M Amran Hossain. | Review | <https://biomedres.us/pdfs/BJSTR.MS.ID.003885.pdf> |
| 10 | ANKLE LIGAMENT RECONSTRUCTION POST-OPERATIVE GUIDELINES. HSS. | Not developed for acute injuries | <https://www.hss.edu/HSSRehabilitationClinicalGuidelines-Ankl.pdf> |
| 11 | Paediatric Clinical Practice Guideline ,NHS. | Age below 18 years old | <https://www.bsuh.nhs.uk/library/wp-content/uploads/sites/8/2020/06/Paediatric-guidelines-ankle-injuries-2019.pdf> |
| 12 | *Physical Therapy Guidelines for Lateral Ankle Sprain.*  *the therapists of MGH Physical Therapy Services* | Not designed using recommended evidence-based guideline methodology | <https://www.massgeneral.org/assets/mgh/pdf/orthopaedics/foot-ankle/pt-guidelines-for-ankle-sprain.pdf> |
| 13 | Rehabilitation Guidelines for  Lateral Ankle Reconstruction.UW health sports rehabilitation | Not developed for acute injuries | <https://bynder.uwhealth.org/m/868637aa7260efb0/original/Rehab-Guideline-Lateral-Ankle-Reconstruction.pdf> |
| 14 | Rehabilitation Protocol for Lateral Ankle Sprain: non-operative management. Massachusetts General Brigham Sports Medicine | Not designed using recommended evidence-based guideline methodology | <https://www.massgeneral.org/assets/mgh/pdf/orthopaedics/sports-medicine/physical-therapy/rehabilitation-protocol-for-ankle-sprain.pdf> |
| 15 | Surgical Guideline for Work-related  Ankle and Foot Injuries. Washington State Department of Labor and Industries Surgical Guideline for Work-related Ankle and Foot Injuries – October 2017 | Not designed using recommended evidence-based guideline methodology | <https://www.lni.wa.gov/patient-care/treating-patients/treatment-guidelines-and-resources/_docs/AnkleFootFinalGuideline081017wcorrection120417.pdf> |
| 16 | Rehabilitation Protocol for Ankle Fracture with ORIF. MGH | Not designed using recommended evidence-based guideline methodology | <https://www.massgeneral.org/assets/mgh/pdf/orthopaedics/sports-medicine/physical-therapy/rehabilitation-protocol-for-ankle-fracture-with-orif.pdf> |
| 17 | *Physical Therapy Guidelines for Total Ankle Arthroplasty. MGH* | Not developed for acute injuries | <https://www.massgeneral.org/assets/mgh/pdf/orthopaedics/foot-ankle/pt-guidelines-total-ankle-arthroplasty-final.pdf> |
| 18 | Rehabilitation Guidelines for Total Ankle Arthroplasty. MGH | Not developed for acute injuries | <https://www.massgeneral.org/assets/mgh/pdf/orthopaedics/sports-medicine/physical-therapy/rehabilitation-protocol-for-total-ankle-arthroplasty.pdf> |
| 19 | *Physical Therapy Guidelines for Peroneal Tendon Repair. MGH* | Not designed using recommended evidence-based guideline methodology | <https://www.massgeneral.org/assets/mgh/pdf/orthopaedics/sports-medicine/physical-therapy/rehabilitation-protocol-for-peroneal-repair.pdf> |
| 20 | *Physical Therapy Guidelines for Ankle Fracture with Surgery.MGH* | Not designed using recommended evidence-based guideline methodology | <https://www.massgeneral.org/assets/mgh/pdf/orthopaedics/foot-ankle/pt-guidelines-ankle-fracture-with-orif-final.pdf> |
| 21 | GUIDELINES FOR PATIENTS FOLLOWING  ANKLE FRACTURE | No clear methodology | <https://www.drgarrettkerns.com/pdfs/guidelines/ankle-fracture.pdf> |
